# Portal Venous Circulating Tumor Cells Undergoing Epithelial-Mesenchymal Transition Exhibit Distinct Clinical Significance in Pancreatic Ductal Adenocarcinoma

**DOI:** 10.3389/fonc.2021.757307

**Published:** 2021-10-28

**Authors:** Yujin Pan, Deyu Li, Jiuhui Yang, Ning Wang, Erwei Xiao, Lianyuan Tao, Xiangming Ding, Peichun Sun, Dongxiao Li

**Affiliations:** ^1^ Department of Hepatobiliary Pancreatic Surgery, Henan Provincial People’s Hospital, People’s Hospital of Zhengzhou University, Zhengzhou, China; ^2^ Zhengzhou Key Laboratory of Minimally Invasive Treatment for Liver Cancer, Henan Provincial People’s Hospital, Zhengzhou, China; ^3^ Henan Provincial Key Laboratory of Hepatobiliary and Pancreatic Diseases, Henan Provincial People’s Hospital, Zhengzhou, China; ^4^ Department of Gastroenterology, Henan Provincial People’s Hospital, People’s Hospital of Zhengzhou University, Zhengzhou, China; ^5^ Department of Gastrointestinal Surgery, Henan Provincial People’s Hospital Zhengzhou, People’s Hospital of Zhengzhou University, Zhengzhou, China

**Keywords:** circulating tumor cell, pancreatic ductal adenocarcinoma, portal venous, prognosis, epithelial-mesenchymal transition

## Abstract

**Background:**

Much importance is attached to the clinical application value of circulating tumor cells (CTCs), meanwhile tumor-proximal CTCs detection has interested researchers for its unique advantage. This research mainly discusses the correlation of portal venous (PoV) CTCs counts in different epithelial-mesenchymal transition status with clinicopathologic parameters and postoperative prognosis in resectable pancreatic ductal adenocarcinoma patients (PDAC).

**Methods:**

PDAC patients (n=60) who received radical resection were enrolled in this research. PoV samples from all patients and peripheral venous (PV) samples from 32 patients among them were collected to verify spatial heterogeneity of CTCs distribution, and explore their correlation with clinicopathologic parameters and clinical prognosis.

**Results:**

CTCs detectable rate and each phenotype count of PoV were higher than those of PV. Patients with recurrence had higher PV and PoV epithelial CTCs (E-CTCs) counts than recurrence-free patients (*P*<0.05). Some unfavourable clinicopathologic parameters were closely related to higher PoV CTCs counts. Multivariate regression analysis demonstrated that PoV mesenchymal CTC (M-CTC)s≥1/5 ml was an independent risk factor for metastasis free survival (MFS) (*P*=0.003) and overall survival (OS) (*P*=0.043).

**Conclusions:**

Our research demonstrated that portal venous was a preferable vessel for CTC test, and patients with PoV M-CTC≥1/5 ml had shorter MFS and OS time in resectable PDAC patients. PoV CTC phenotype detection has the potential to be a reliable and accurate tool to identify resectable PDAC patients with high tendency of postoperative metastasis for better stratified management.

## Introduction

Pancreatic ductal adenocarcinoma (PDAC) remains one of the most aggressive malignancies with high metastatic tendency to distant organs (1), and it has been projected to be the second most lethal tumor by 2030 (2). A retrospective study pointed that nearly half of 957 PDAC patients had suffered tumor recurrence or metastasis within 1 year after surgery, including liver metastasis (33.8%) and lung metastasis (8.5%), though they had already received radical surgery (3). Cancer management strategy would benefit a lot from the accurate prognostic indicator in order to better stratify patients (4, 5). While the prognosis of PDAC is highly unpredictable, mainly due to the absence of precise and timely prognosis indicators (6).

Increasing evidence demonstrated that utilizing the circulating tumor cells (CTCs) count to early assess the postoperative prognosis can be considered as an efficient method in some solid tumors (5, 7–9). To date, research involving CTCs detection has mainly focused on peripheral blood samples with low detectable rates and detectable enumeration (7, 9). It is worth noting that researchers have utilized tumor-proximal liquid biopsy to overcome the mentioned limitations with the enhancement of detectable rates and the enumeration of CTCs (7, 9–11). In this research, we collected portal venous blood and peripheral venous blood for CTCs phenotypes detection to verify the value of tumor-proximal liquid biopsy.

Epithelial-mesenchymal transition (EMT) and its intermediate states have already been acknowledged as crucial drivers of tumor progression, though there is still far from a consensus on the significance of each EMT phenotype for prognosis (12, 13). Recently, exploring the correlation between CTCs in different EMT statuses and prognosis has attracted increasing attention (14–16). In this study, Canpatrol CTC detection technology was used to enrich and identify CTCs in different EMT statuses and then routinely classified them into three typical subgroups according to the expression of fluorescence signals: entirely epithelial-CTCs(E-CTCs), entirely mesenchymal-CTCs (M-CTCs), and hybrid phenotype CTCs (H-CTCs). Furthermore, we classified the H-CTC into E>M, E≈M, and E<M subtypes. The main aim of this study is to identify the correlation between the portal venous CTCs subtypes count with clinicopathological parameters and prognosis to verify the clinical application value of tumor-proximal liquid biopsy in resectable PDAC patients.

## Patients and Methods

### Patients

This research was carried out at the Department of Hepatobiliary and Pancreatic Surgery, People’s Hospital of Zhengzhou University (Zhengzhou, China). From August 2018 to September 2020, 60 PDAC patients receiving radical surgery were enrolled. The inclusion criteria were as follows: (a) radical resection confirmed by postoperative pathology; (b) PoV blood samples collecting was prior to specimen separation intraoperation; (c) definite pathological diagnosis of PDAC. The exclusion criteria were as follows: (a) previously treated with anticancer therapy before surgery, including radiotherapy, immunotherapy and chemotherapy; (b) patients with incomplete clinicopathological data; (c) patients with distant metastasis or others primary tumor. Ultimately, sixty patients met the inclusion criteria and were enrolled in this study. This study is consistent with the guidelines of the Declaration of Helsinki and approved by ethics committee of Henan Province People’s hospital. Informed consent was obtained from each participant.

### Portal Venous and Peripheral Venous Blood Samples

**Figure 1:**
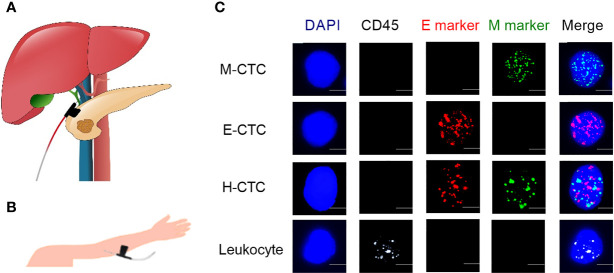
Representative images of PoV, PV blood samples collection and typical multifluorescence signals of CTCs and leukocytes. **(A)** Collecting the portal venous blood sample. **(B)** Peripheral vessel blood sample was obtained by puncturing the cubital vein. **(C)** Based on mRNA sequence staining technology, the nuclei were stained with DAPI (blue), the epithelial markers (EpCAM and CK8/18/19) are indicated by red dots, the mesenchymal markers (Vimentin and Twist) are indicated by green dots, and the hybrid CTCs contain epithelial markers and mesenchymal markers are indicated by red and green dots. Leukocyte marker (CD45) is indicated by white dots. Scale bar = 5μm.

Portal venous (PoV) blood sample (5ml) collection was performed prior to specimen separation during surgery (**Figure 1A**). Peripheral blood (PV) samples (5ml) were obtained from the cubital vein before surgery (**Figure 1B**). Then, these blood samples were placed in ethylenediaminetetraacetic acid (EDTA) vacutainers for the CTC phenotype detection.

### CTCs Enrichment and Identification

The Canpatrol™ CTCs detection platform (Sur Exam, Guangzhou, China) had been introduced by previous reports (5, 17, 18). Firstly, CTCs were isolated by a filtration system containing a membrane with 8-μm diameter pores (Sur Exam, Guangzhou, China). The nuclei were stained by4',6-diamidino-2-phenylindole (DAPI). RNA-*in situ* hybridization technology was used to identify the CTC phenotypes targeting different mRNA sequences that encode epithelial biomarkers (EpCAM, CK8/18/19), mesenchymal biomarkers (Vimentin and Twist), and the leukocyte biomarker CD45. CTCs phenotypes were analyzed with a fluorescent microscope, the epithelial CTCs (E-CTCs) were stained with red fluorescence, mesenchymal CTCs (M-CTCs) were stained with green fluorescence and hybrid CTCs (H-CTCs) were stained with both red and green fluorescence. Besides, leukocytes were stained with white fluorescence (**Figure 1C**).

### Clinical and Pathologic Characteristic

Clinicopathological data from 60 PDAC patients were collected, including age, sex, hepatitis, diabetes, preoperative serum CA19-9 level and postoperative serum CA19-9 level (referring to the first postoperative review result), tumor location (head and neck *vs* body and tail), degree of tumor differentiation (medium and poorly differentiated *vs* well differentiated), TNM stage (referring AJCC cancer staging manual, 8th edition) (19), nerve invasion, tumor size, surgical method, PoV and PV CTCs subtype counts.

### Follow Up

The postoperative follow-up mainly focuses on the disease progression, including recurrence, metastasis, death until to January 2021. According to the guidelines of PCCA, patients are recommended to undergo a comprehensive examination for status assessment every 3 months in the first year, every 3 to 6 months during the second to third years, and then every 6 months (20). The length of the recurrence free survival (RFS)/metastasis free survival (MFS)/overall survival (OS) was measured from the date of surgery until the date of recurrence/metastasis/death occurring.

Postoperative tumor progression was stratified into two mutually exclusive categories: local recurrence and distant metastasis and they were judged mainly by imaging examination. If a patient had diagnosed with both local recurrence and distant metastasis at a follow-up, then he was classified into the metastasis group for metastatic lesions can represent a more lethal progression.

### Statistical Analysis

SPSS 21.0 and GraphPad Prism 8.0 software were used for data analysis. E-CTCs, M-CTCs, H-CTCs and T-CTC counts were used to investigate the prognosis assessment value and the correlation with clinicopathological parameters. Receiver operating characteristic curve (ROC) was used to determine the cut-off value of each CTCs subtype counts on different clinical outcomes. Then, we divided these patients with different CTC subtype count into different groups.

Continuous variables are presented as the median with inter-quartile range (IQR). CTCs count differences were compared by the nonparametric Mann–Whitney U test and/or the Kruskal-Wallis test. The Kaplan–Meier method with the log-rank test was used to assess the differences between the different groups. Univariate and multivariate factor analyses of prognosis-related factors were conducted using a Cox regression model to identify independent predictors. *P*<0.05 means statistically significant.

## Results

### Patients Characteristics

Sixty patients were enrolled in this study, including 34 men and 26 women with a median age of 56.5 (range: 30–83 years old). The number of patients in I stage, II stage, and III stage of TNM stage were 16, 35, 9 respectively. In addition, PV blood samples were collected from 32 patients among them. Although it was an invasive operation to obtain portal venous blood, we had not observed immediate or delayed complications from portal vein draws including hematoma formation or gastrointestinal bleeding. With a median follow‐up duration of 15 months (range: 3–29 months), 11 (18.3%) patients experienced local recurrence, and metastasis occurred in 30.0% (18/60), including 9 liver only metastases, 4 lung only metastases, 3 cases of multiple sites metastases, beside 3 patients had been diagnosed with both recurrence and metastatic. By the last follow-up, 17 patients had died due to tumor progression.

### Spatial Heterogeneity of CTCs Distribution

**Figure 2:**
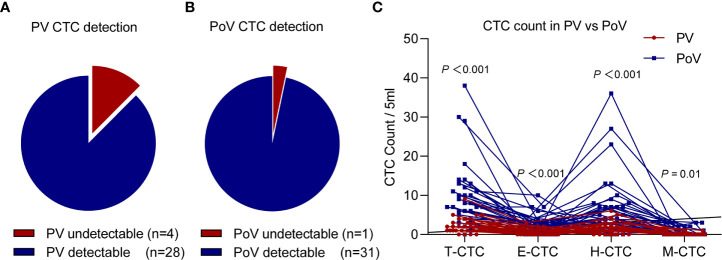
The CTCs count paired comparison in PV and PoV samples (n=32). The detectable rate of T-CTCs in the PV sample **(A)** was lower than that of the PoV sample **(B)** (87.5% *vs* 96.9%). **(C)** Each CTCs phenotype (T-CTC, E-CTC, H-CTC, M-CTC) count of the PoV sample were higher than those of the PV sample.

Thirty-two patients were implemented both PoV and PV CTCs phenotype tests, and we found that the T-CTC detectable rate in PV was 87.5% (**Figure 2A**), while the PoV T-CTC detectable positive ratio was high to 96.9% (**Figure 2B**). Paired comparison of CTCs phenotype counts in different vessels of 32 patients, we found the count of T-CTC and three kinds of subtypes of CTC in PoV were all significantly higher than in PV (**Figure 2C**) (*P*<0.05).

To further investigate the correlation between the PoV and PV CTC counts, we performed a linear regression analysis and found that the counts of T-CTCs, H-CTCs, E-CTCs per 5ml of PoV were significantly correlated with the count of PV (*R^2^* = 0.349, *P <* 0.001; *R^2^* = 0.335, *P <* 0.001; *R^2^ = 0.135, P =* 0.039; respectively) (**Supplementary Figures 1A–C**), while no significant association was observed in the count of M-CTCs per 5 mL of PoV and PV sample (*R^2^ <* 0.001, *P* = 0.973) (**Supplementary Figure 1D**).

### PV CTCs Phenotype and Their Prognostic Significance

During the follow-up (median months=15, range 3 to 29), five patients (15.6%) suffered recurrence, eleven patients (34.5%) presented with tumor metastasis, and eleven patients (34.5%) died from tumor-related causes. The median counts of T-CTCs, E-CTCs, H-CTCs and M-CTCs were 2, 0.5, 1, and 0, respectively.

**Figure 3:**
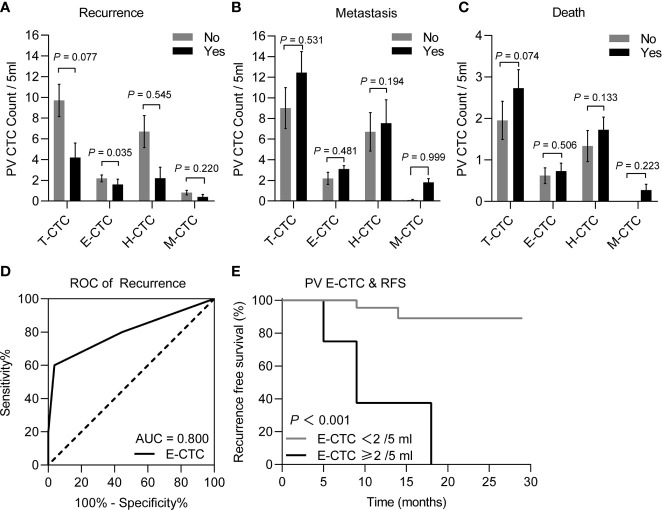
Comparison of different CTC phenotype counts of peripheral blood samples between the PDAC patients (n=32) with different postoperative prognosis, including recurrence or recurrence-free **(A)**, metastasis or metastasis-free **(B)**, and death or survival **(C)**, through the Mann–Whitney U test, the results demonstrated that the recurrence patients (n=5) had significantly higher E-CTC counts than the recurrence-free patients (n=27) (*P* < 0.05). **(D)** ROC curves for PV E-CTC (cut-off = 2 CTCs/5 ml, AUC = 0.800 95% CI 0.621–0.920; *P* = 0.032) regarding on recurrence. **(E)** Kaplan-Meier RFS stratified with respect to the E-CTC cut-off value of 2/5 ml for PDAC patients, the curve showed patients with PV E-CTC ≥ 2/5ml had a significantly shorter RFS than patients with PV E-CTC < 2/5ml (*P* = 0.0002).

To evaluate the prognostic value of the PV CTC phenotype count in PDAC, we compared the count differences of T-CTC and CTCs subtype among patients with different outcomes including recurrence, metastasis, death. We had not noticed a correlation between PV CTCs subtype counts with survival and metastasis events (**Figures 3B, C** **)**. However, we found that recurrent patients had higher E-CTC counts than recurrence-free patients (*P*<0.05) (**Figure 3A** **)**. ROC curve analysis exhibited that E-CTC≥2/5 ml means a higher risk of tumor recurrence (*P*=0.02) (**Figure 3D**), and Kaplan–Meier curve analysis shown that patients with high PV E-CTC (E-CTC ≥ 2/5 ml) counts had significantly shorter RFS than patients with low E-CTC counts (*P*=0.002) (**Figure 3E**).

### PV CTCs Count and Prognosis in Patients Received Adjuvant Chemotherapy

Peripheral blood CTCs of 18 unresectable advanced PDAC patients were also collected before and after the first cycle chemotherapy and chemotherapy mainly adopts gemcitabine - based monotherapy or combination regimens. Comparing the PV CTCs subtype count difference in resectable and unresectable PDAC patients before chemotherapy, we noticed that unresectable patients had significantly higher H-CTCs than resectable patients (*P*=0.035) (**Supplementary Figure 2A**). Besides, Kaplan-Meier analysis shown that there was no significantly difference on overall survival in patients with increased or non-increased T-CTCs/E-CTCs/H-CTCs/M-CTCs (**Supplementary Figures 2B–E**) (*P=*0.915, *P=*0.149, *P=*0.505, *P=*0.164, respectively).

### PoV CTCs Count With Clinicopathologic Parameters and Prognosis

We obtained PoV blood samples for CTC phenotype test from 60 patients. Fifty-eight patients (96.7%) had detectable T-CTC in the PoV, and the median counts of T-CTCs, E-CTCs, H-CTCs and M-CTCs were 7, 2, 4, and 0, respectively. During the follow-up (median months=15, IQR 10.25 to 18), eleven patients (18.3%) presented with disease recurrence, eighteen patients (30%) presented with tumor metastasis, and seventeen patients (28.3%) died due to tumor-related causes.

The correlations between the clinicopathologic variables and the PoV CTC phenotype count of the 60 PDAC patients are listed in **Table 1**. The Mann-Whitney U test was used to compare the correlation between CTCs subtype counts and the clinicopathologic variables and outcomes. In this research, we noticed a correlation of the T-CTC count with an abnormal postoperative CA19-9 level and tumor recurrence; a correlation of the E-CTC count with a postoperative abnormal CA19-9 level, T stage and tumor recurrence; a correlation of the H-CTC count with a postoperative abnormal CA19-9; a correlation of the M-CTC count with lymph node invasion, tumor differentiation degree and tumor metastasis (*P*<0.05) (**Table 1**).

**Table 1:** Correlation between PoV CTC phenotype count and baseline characteristics.

Variables	n	T-CTC	*P*	E-CTC	*P*	H-CTC	*P*	M-CTC	*P*
		Median (IQR)		Median (IQR)		Median (IQR)		Median (IQR)	
Gender			0.487		0.142		0.742		0.061
Male	34	7.5 (4.75-13.25)		2 (1-4.25)		4 (1.75-8)		0 (0-1.25)	
Female	26	7 (3-13.25)		2 (0.75-3)		4.5 (2-9.5)		0 (0-0)	
Age (years)			0.470		0.234		0.829		0.452
≤65	38	7 (4-14)		2 (1-3.25)		4 (2-8.5)		0 (0-1)	
>65	22	7.5 (2-13)		1.5 (0-4.25)		5 (1.75-8.25)		0 (0-1)	
Diabetes			0.336		0.343		0.099		0.942
No	46	7 (3.75-13)		2 (1-5)		4 (1-7.5)		0 (0-1)	
Yes	14	9.5 (4.75-15)		2 (1-3)		7 (3-13)		0 (0-1.25)	
Hepatitis			0.959		0.925		0.795		0.902
No	51	7 (4-13)		2 (1-4)		5 (2-8)		0 (0-1)	
Yes	9	7 (3.5-13.5)		2 (1-3.5)		3 (2.5-9)		0 (0-2)	
Pre-op CA19-9 (u/ml)			0.087		0.853		0.038		0.335
≤37	23	6 (3-10)		2 (1-4)		3 (1-6)		0 (0-1)	
>37	37	8 (5-14)		2 (1-3.5)		6 (3-9.5)		0 (0-1.5)	
Post-op CA19-9 (u/ml)			0.013*		0.030*		0.012*		0.339
≤37	40	6.5 (3-11)		2 (0.25-3)		3 (1-7)		0 (0-1)	
>37	20	11.5 (6.25-18.75)		3 (1.25-5.75)		7 (3.25-15.25)		0 (0-0.75)	
T stage			0.061		0.006*		0.514		0.367
T1-T2	30	6.5 (3-10.5)		2 (0-2)		4 (2-7)		0 (0-1)	
T3	30	10 (4.75-17.25)		3 (1-5)		5.5 (1-11.5)		0 (0-2)	
Tumor location			0.242		0.099		0.859		0.349
Head、neck	29	9 (4-16)		3 (1-4.5)		5 (1-10.5)		0 (0-1.5)	
Body、tail	31	7 (3-13)		2 (1-3)		4 (2-8)		0 (0-0)	
Lymph node invasion			0.141		0.810		0.207		0.014*
N0	30	6.5 (3-11.5)		2 (0.75-3.25)		3.5 (1.75-7.25)		0 (0-0)	
N1-N2	30	8.5 (4-14.75)		2 (1-4)		5.5 (2.75-10)		0 (0-2.25)	
TNM stage 8th AJCC			0.852		0.858		0.795		0.710
IA-IIB	51	7 (4-13)		2 (1-3)		4 (2-8)		0 (0-1)	
III	9	8 (3-21)		2 (0.5-4.5)		3 (1-15)		0 (0-1.5)	
Nerve invasion			0.064		0.128		0.240		0.225
No	34	7 (3-10)		2 (0-3)		4 (2-7)		0 (0-1)	
Yes	26	11 (4.75-17.25)		2.5 (1-4.25)		6.5 (1.75-13)		0 (0-2)	
Differentiation			0.125		0.201		0.375		0.028*
High	36	7 (3-11.5)		2 (1-3)		3.5 (2-7)		0 (0-0)	
Middle and low	24	10.5 (4.5-14)		2.5 (1-5)		5 (2.25-10.75)		0.5 (0-2)	
Recurrence			0.025*		0.021*		0.067		0.309
No	49	7 (4-11.5)		2 (1-3)		4 (2-7)		0 (0-1)	
Yes	11	17 (6-23)		5 (1-7)		11 (3-18)		0 (0-0)	
Metastasis			0.068		0.075		0.616		<0.001*
No	42	7 (3-11)		2 (1-3)		4 (2-8)		0 (0-0)	
Yes	18	12 (5.5-15)		3 (2-4.25)		5 (1.5-10)		2 (0.75-3)	
Death			0.278		0.070		0.439		<0.001*
No	43	7 (3-13)		2 (1-3)		4 (2-9)		0 (0-0)	
Yes	17	12 (5-13.5)		3 (1.5-5)		4 (0.5-7.5)		1 (0-3)	

Pre-op, preoperative; post-op, postoperative.

Bold values indicate that the results were statistically significant.

**Figure 4:**
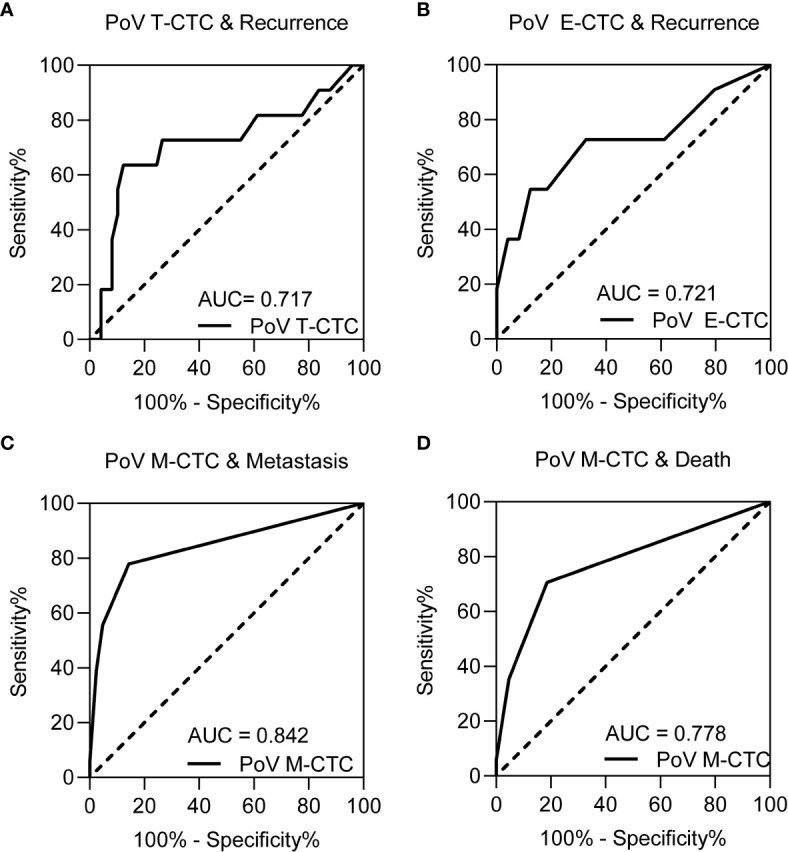
The ROC curve for **(A)** PoV T-CTCs, **(B)** PoV E-CTCs on recurrence prediction, **(C)** PoV M-CTCs for metastasis prediction and **(D)** PoV M-CTCs for death prediction. The result of sensitivity, specificity and cut-off value of each ROC curve are depicted in **Table 2**.

**Figure 5:**
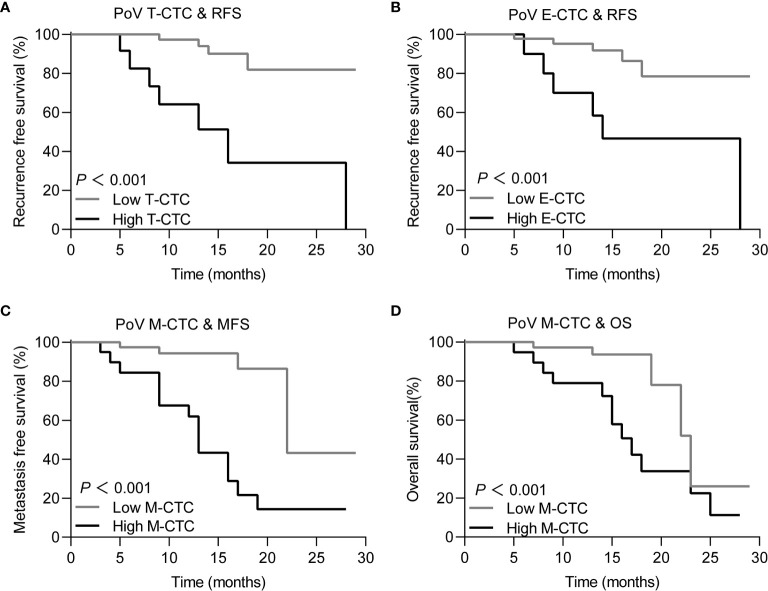
Kaplan-Meier curves for PDAC patients with different prognosis in high/low CTC subtype count group for **(A)** T-CTC, for patients with high T-CTCs (≥ 14/5 ml) *vs* low T-CTC (<14/5 ml) and the mean RFS was 26.44 months (95%CI 24.07-28.83) *vs* 16.51 (95%CI 10.17 - 22.85). **(B)** E-CTC, for patients with high E-CTCs (≥ 5/5 ml) *vs* low E-CTC (<5/5 ml) and the mean RFS was 25.84 month (95%CI 23.268- 28.416) *vs* 18.51 (95%CI 12.106 - 24.927). **(C)** M-CTC, for patients with high M-CTCs (≥ 1/5 ml) *vs* low M-CTC (<1/5 ml) and the mean MFS was 14.19 month (95%CI 10.77 - 17.61) *vs* 23.78 (95%CI 19.207 - 28.368). **(D)** M-CTC, for patients with high M-CTCs (≥ 1/5 ml) *vs* low M-CTC (<1/5 ml) and the mean OS was 17.28 months (95% CI 13.91 to 20.65) *vs* 22.87 months (95% CI 19.51 - 26.22).

Based on the correlation between the clinical outcome and the CTC phenotype counts, ROC curves were used to determine the cut-off value of the CTC phenotype counts **Figures 4A–D**. The sensitivity, specificity and cut-off value are depicted in **Table 2**. According to tumor recurrence or recurrence-free, the cut-off values of the T-CTC and E-CTC counts were 14/5 ml and 5/5 ml, respectively. The cut-off value of M-CTC to describe postoperative metastasis was 1/5 ml, and M-CTC≥1/5 ml was also an unfavourable factor of OS (**Figure 5**).

**Table 2:** ROC curves of PoV CTCs phenotype counts on postoperative progression.

Clinical outcome	CTC phenotype	Cut-off value	Sensitivity	Specificity	AUC	*P* value
Recurrence	T-CTC	14	63.6	87.8	0.717	0.035*
Recurrence	E-CTC	5	54.55	87.76	0.721	0.032*
Metastasis	M-CTC	1	77.78	85.71	0.842	<0.001*
Death	M-CTC	1	70.60	81.40	0.778	<0.001*

Bold values indicate that the results were statistically significant.

Based on the cut-off value, PoV CTC subtype counts were divided into high or low groups. Then, Kaplan–Meier analysis was used to compare the prognostic differences among the CTC counts in different groups. The Kaplan–Meier curves showed that PoV T-CTC≥14/5 ml predicts a shorter RFS (*P*<0.05), PoV E-CTC≥5/5 ml also predicts a shorter RFS (*P*<0.05), while PoV M-CTC≥1/5 ml predicts a shorter MFS (*P*<0.05), and a shorter OS (*P*<0.05)

### PoV H-CTC Count to Predict the Postoperative Prognosis

**Figure 6:**
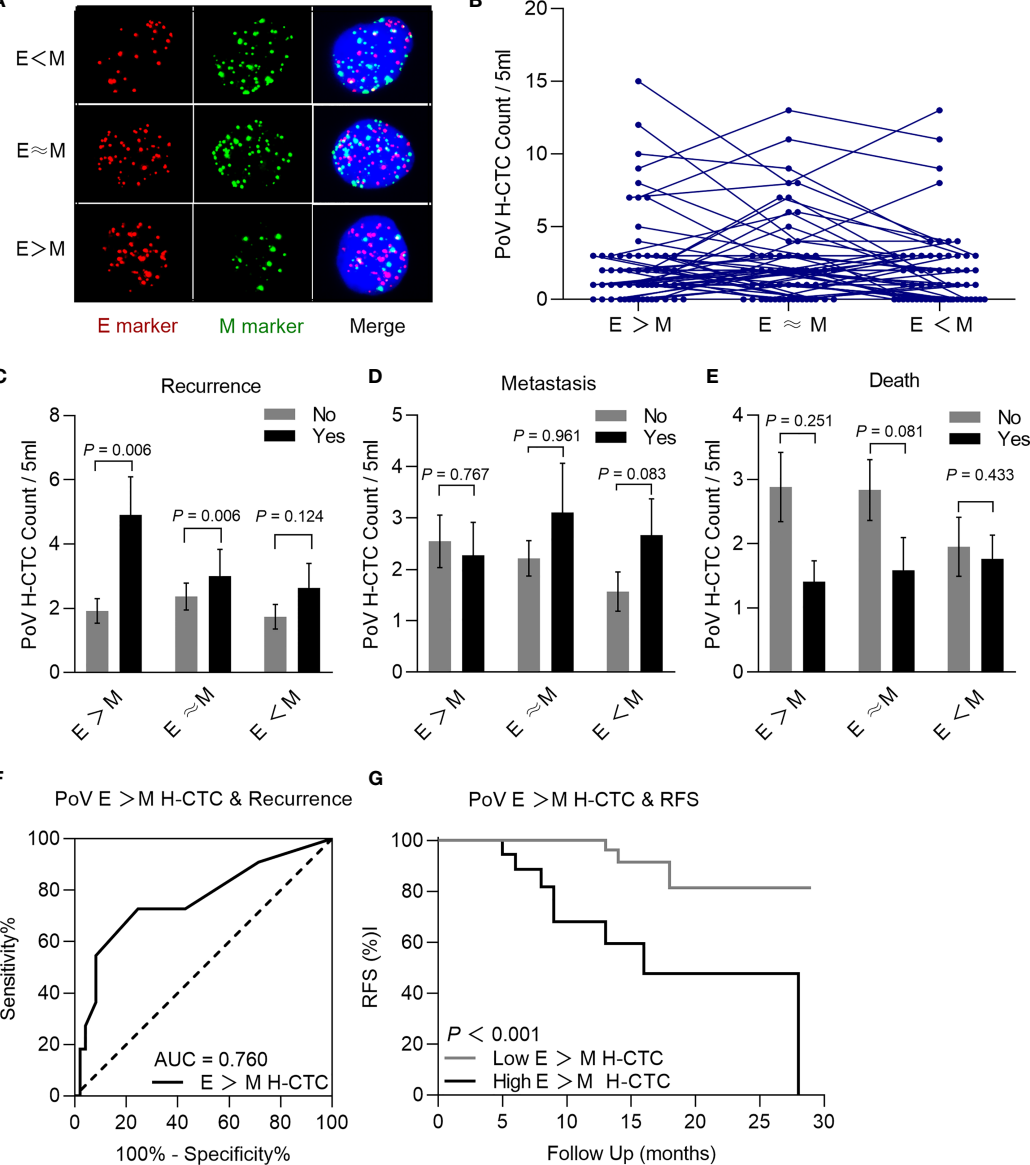
Typical multi-fluorescence signals of hybrid CTCs subtypes and correlation with prognosis. **(A)** Epithelial predominant (E > M) hybrid CTC, intermediate (E≈ M) hybrid CTC, or mesenchymal predominant (E<M) hybrid CTC. **(B)** Each H-CTC subtype count in patients (n=60). Comparison of PoV H-CTC subtype counts with prognosis, recurrence **(C)**, metastasis **(D)**, and death **(E)**, the result demonstrated that recurrence patients had significantly higher E > M H-CTC counts than recurrence-free patients (*P*<0.05). **(F)** ROC curve was used to determine that the cut-off value of E > M H-CTC count was 3/5 ml (AUC = 0.760, sensitivity = 72.7%, specificity =75.5%, *P*=0.004). **(G)** Kaplan–Meier analysis showed that patients with E>M H-CTC ≥ 3/5ml had significantly shorter RFS than patients with E>M H-CTC<3/5ml (*P* < 0.001).

A scholar had also adapted the Canpatrol™ CTC platform, then divided the CTCs into five phenotype with different plastic and reversible phenotypes (21), referring this division criteria, the H-CTCs were further classified into three subtypes as epithelial predominant (E>M), intermediate (E≈M), or mesenchymal predominant (E<M) according to the signal numbers of epithelial or mesenchymal biomarker (**Figure 6A**). H-CTCs are the main component of CTCs in both PV and PoV; interestingly, E ≈ M is also the most abundant subtype of H-CTCs (**Figure 6B**). We noticed a correlation between PoV E>M H-CTCs with postoperative recurrence, besides we had not found correlations between the other CTCs phenotypes with postoperative metastasis and survival (**Figures 6C–E**). The ROC curve results showed that the cut-off value of E> M H-CTC counts for recurrence assessment was 3/5 ml (**Figure 6F**). The Kaplan–Meier curves demonstrated that E>M H-CTC≥3/5 ml means a shorter RFS **(** **Figure 6G** **).**

### Univariate and Multivariate Regression Analysis of Postoperative Prognosis

To identify the influence of baseline characteristics and particular PoV CTC phenotype count on the resectable PDAC postoperative prognosis. We noticed that postoperative CA19-9>37 U/ml, high T stage, PoV E>M H-CTC≥3/5 ml, PoV T-CTC≥14/5 ml, and PoV E-CTC≥5/5 ml were significantly associated with RFS (all *P*<0.05), while lymph node invasion and PoV M-CTC≥1/5 ml were significantly associated with MFS (all *P*<0.05) by univariate Cox regression. However, M-CTC≥1/5 ml and lymph node invasion were both associated with significantly shorter OS (all *P*<0.05) (**Table 3**). Next, the multivariable analysis revealed that postoperative CA19-9>37 U/ml was a significant independent predictor of RFS (95CI 1.237–40.908; *P*=0.028); lymph node invasion (95%CI 1.286–19.546; *P*=0.020) and PoV M-CTC≥1/5 ml (95%CI 1.893–24.396; *P*=0.003) were significantly associated with shorter MFS; and PoV M-CTC≥ 1/5 ml (95%CI 1.03–9.27; *P*=0.043) was significantly associated with shorter OS (**Table 4**).

**Table 3:** Univariate Cox regression analysis for postoperative progression.

Variables	Recurrence Univariate Analysis		Metastasis Univariate Analysis		Death Univariate Analysis	
	HR (95% CI)	*p*	HR (95% CI)	*p*	HR (95% CI)	*p*
Gender: Male *vs* Female	1.347 (0.410-4.425)	0.623	0.779 (0.300-2.020)	0.607	0.533 (0.187-1.525)	0.241
Age (years): ≤65 *vs* >65	1.380 (0.419-4.549)	0.596	0.520 (0.171-1.586)	0.251	0.937 (0.326-2.689)	0.903
Diabetes: no *vs* yes	0.726 (0.153-3.442)	0.687	1.488 (0.547-4.048)	0.436	1.482 (0.533-4.115)	0.451
Hepatitis: no *vs* yes	1.978 (0.419-9.351)	0.389	1.135 (0.257-5.017)	0.867	2.762 (0.737-10.357)	0.132
Pre-op CA19-9 (u/ml): ≤37 *vs*>37	1.294 (0.377-4.439)	0.682	2.203 (0.722-6.728)	0.165	1.329 (0.461-3.827)	0.540
Post-op CA19-9 (u/ml):≤37 *vs*>37	9.432 (2.026-43.916)	0.004*	0.423 (0.122-1.464)	0.175	0.540 (0.173-1.688)	0.396
T stage: T1-T2 *vs* T3	5.675 (1.222-26.355)	0.027*	1.824 (.699-4.755)	0.219	1.464 (0.548-3.906)	0.447
Tumor location Head、neck *vs* Body、tail	1.081 (0.304-3.842)	0.904	0.439 (0.170-1.138)	0.090	0.615 (0.236-1.603)	0.320
Total laparoscopic: no *vs* yes	2.128 (0.564-8.037)	0.265	0.555 (0.216-1.423)	0.220	1.039 (0.390-2.771)	0.939
Lymph node invasion: N0 *vs* N1-N2	1.146 (0.347-3.785)	0.823	6.355 (1.829-22.086)	0.004*	3.455 (1.112-10.733)	0.032*
TNM stage: IA-IIB *vs* III	1.706 (0.362-8.049)	0.500	1.624 (0.528-4.998)	0.398	1.687 (0.537-5.295)	0.371
Nerve invasion: no *vs* yes	2.628 (0.731-9.446)	0.139	2.035 (0.769-5.380)	0.152	1.920 (0.706-5.225)	0.202
Differentiation: High *vs* Middle and low	2.940 (0.849-10.185)	0.089	3.253 (1.216-8.704)	0.019*	2.070 (0.757-5.661)	0.156
PoV E>M H-CTC (per 5mL): <3 *vs* ≥ 3	6.867 (1.802-26.161)	0.005*	1.318 (0.492-3.528)	0.583	0.935 (0.327-2.671)	0.900
PoV T-CTC (per 5mL): <14 *vs* ≥14	8.075 (2.308-28.248)	0.001*	1.698 (0.596-4.837)	0.321	1.016 (0.325-3.183)	0.978
PoV E-CTC (per 5mL): <5 *vs* ≥ 5	4.989 (1.490-16.707)	0.009*	1.253 (.410-3.828)	0.693	1.138 (0.385-3.365)	0.815
PoV M-CTC (per 5mL): <1 *vs* ≥ 1	0.485 (0.103-2.274)	0.358	7.704 (2.527-23.492)	<0.001*	3.963 (1.371-11.455)	0.011*

Variables which had a P value < 0.05 in univariable analysis was included in the multivariable analysis.

Bold values indicate that the results were statistically significant.

**Table 4:** Multivariate Cox regression analysis for postoperative progression.

Variables	Recurrence multivariate analysis		Metastasis multivariate analysis		Death multivariate analysis	
	HR (95% CI)	*p*	HR (95% CI)	*p*	HR (95% CI)	*p*
Post-op CA19-9 (u/ml):≤ 37 *vs*>37	7.11 (1.23-40.90)	0.028*	NA.		NA.	
T stage: T1-T2 vs T3	4.37 (0.75-25.55)	0.10	NA.		NA.	
Lymph node invasion: N0 *vs* N1-N2	NA.		5.01 (1.28-19.54)	0.020*	2.531 (0.78-8.18)	0.12
Differentiation: High *vs* Middle and low	NA.		0.67 (0.20-2.27)	0.52	NA.	
PoV E>M H-CTC (per 5ml): <3 *vs* ≥ 3	1.22 (0.11-13.08)	0.870	NA.		NA.	
PoV T-CTC (per 5ml): <14 *vs* ≥ 14	3.84 (0.48-30.28)	0.202	NA.		NA.	
PoV E-CTC (per 5ml): <5 *vs* ≥ 5	0.58 (0.094-3.64)	0.56	NA.		NA.	
PoV M-CTC (per 5ml): <1 *vs* ≥ 1	NA.		6.795 (1.89-24.39)	0.003*	3.100 (1.03-9.27)	0.043*

NA, no application.

Bold values indicate that the results were statistically significant.

## Discussion

In this study, 88.2% (15/17) of PDAC patients died due to postoperative metastasis with a median follow-up duration of 15 months. Tumor metastasis is an extremely aggressive characteristic of tumors and is the mainly cause of tumor related death of more than 90% of patients with malignant tumors (22). The most common metastasis organ of PDAC patients is the liver due to its unique anatomical features and microenvironment (1, 4, 7). Identifying patients with high tendency of postoperative metastasis or recurrence is of the utmost importance, and it could contribute to the development of novel treatment model and management strategies for PDAC patients (6, 7).

In EMT (epithelial–mesenchymal transition) process, tumor cells acquire some properties of mesenchymal cells which promote tumor cell migrate and invade (16, 23). However, whether EMT proceeds through intermediate states and, if so, how many intermediate steps exist in this transition, how plastic and reversible these intermediate states are, and what the implications of these different EMT status are for clinical applications (24). EMT and its different intermediate status have recently been noticed as crucial drivers of tumor progression and metastasis (16, 24, 25). In light of recent research demonstrated that the CTC count could be used as a prognostic biomarker, and a higher CTCs count may predict an unfavourable prognosis in malignant patients (26–30). In this research, we used the Canpatrol™ CTC filtration platform to detect CTCs in different EMT statuses intend to explore their prognostic assessment value. The results of the PV CTC and PoV CTC subtype counts confirmed a correlation between high PV E-CTC, PoV E-CTC, and PoV E>M H-CTC counts with postoperative recurrence, but they were not significant independent risk factors of recurrence, while high M-CTC counts were significant independent risk factors for postoperative metastasis and death.

EMT is rather a binary process, and epithelial CTCs gradually lose polarity and intercellular adhesion, but gain increased migratory and invasive properties in this process (5, 30). CTCs have been reviewed as the seeds of tumor metastasis, and previous reports had noted that mesenchymal CTCs with an elongated shape were closely related to the tumor metastasis cascade (24, 31). Although we are far from reaching a consensus on the detailed mechanisms, some researchers have already demonstrated that M-CTCs play an essential role in tumor progression, especially the distant metastasis process (5, 30, 32, 33). In this research, we also noticed a correlation between PoV E>M H-CTC, PoV E-CTCs counts with RFS. The proliferation capability of E-CTCs and early hybrid CTCs may lead to tumor relapse may be due to their unique self-seeding effect (34).

The count of each PoV CTC subtype varied in patients with different clinicopathological factors. In our study, PoV M-CTCs counts were linked to lymph node invasion and tumor differentiation degree. Besides, PoV T-CTCs were closely related to the postoperative CA19-9 level, the E-CTCs count were closely related to the postoperative CA19-9 level and high T stage, meanwhile the H-CTC count was also related to the postoperative CA19-9 level. The above results demonstrated that CTCs with different statuses might not be a separate indicator of tumor progression and they also had a closely association with the common clinical and pathological characteristics.

A previous study had reported that surgical operation could cause the increasing of PoV CTC count (35) and we total agree with this opinion, so we collected PoV sample prior to specimen separation during surgery. Paired analysis of the CTCs counts and detectable rate in the PV and PoV blood sample, we noticed that the CTCs detectable rate and CTCs count in PoV were both significantly higher than in PV and it is consistent with several previous reports that demonstrated the spatial heterogeneity of CTCs distribution (4, 7, 9). Besides, we also noticed a correlation between the PoV and PV CTCs counts in the count of T-CTC, H-CTC and M-CTC, and this phenomenon indirect the peculiar property of M-CTC.

Detecting CTCs in peripheral blood of PDAC was still challenging due to hepatic filtration and technical limitations which may limit its clinical application value (7, 36). Notably, abundant previous research had demonstrated that tumor-proximal liquid biopsy can enhance the diagnostic and prognosis assessment performance of CTCs in vessels closer to the tumor (4, 7, 9, 37). Portal veins are the main veins that drain blood from the pancreas to other organs, which may be related to the high frequency of liver metastasis from pancreatic cancer (38). portal venous as the main drain tube of pancreas with abundant CTCs and the promising clinical application value of portal venous CTCs test had been demonstrated by previous studies (4, 7, 9, 37). Recently, ultrasound-guided (9) and endoscopic ultrasound-guided (39, 40) fine-needle aspiration have gradually been used to obtain portal venous blood in advanced PDAC patients. However, portal vein puncture is an invasive approach with the possibility of bleeding、infection、thrombotic、tumor cell spread. besides it is also a challenge to recognize and obtain portal venous blood during surgery, especially in the vision of laparoscopic which may extend the operation duration.

Several reports had demonstrated that peripheral CTCs could be used to assess prognosis and the effect of chemotherapy in cancer patients (41–44). In addition, monitoring the dynamically change of peripheral CTC to assess the adjuvant therapy and neoadjuvant therapy effect could help to timely identify ineffective treatment and avoid unnecessary costs (45). In this research, we monitored the peripheral blood CTCs of 18 unresectable advanced PDAC patients before and after the first cycle chemotherapy, but we had not noticed the significantly difference on the overall survival time in patients with increased or non-increased CTCs regardless of the subtype which may related to the low counts of PV CTC and small research cohorts.

Some limitations exist in this research, including the small cohort size, short follow-up time, and single centre research. In addition, other unpredictable factors may also influence the final results. In this study, we used Canpatrol™ technology to divide the EMT procedure into five stages, but the detailed procedure and property differences had not reached a consensus. Moreover, we should recognize that different detection methods for CTCs may lead to inconsistent results.

In conclusion, we have identified the spatial heterogeneity of the CTC distribution, and portal veins may be a better vessel for CTC phenotype testing to assess the PDAC prognosis than peripheral vessels. In addition, high PoV M-CTC counts are significant independent risk factors for postoperative metastasis and survival. Therefore, the PoV CTC phenotype test with the potential to be developed into an accurate and reliable biomarker to guide treatment decisions and patient stratified management.

## Data Availability Statement

The raw data supporting the conclusions of this article will be made available by the authors, without undue reservation.

## Ethics Statement

The studies involving human participants were reviewed and approved by Henan Provincial People’s Hospital. The patients/participants provided their written informed consent to participate in this study.

## Author Contributions

YP and DYL performed the literature search and manuscript drafting. JY, XD, and NW conducted data collection. YP, EX, and LT conducted statistical processing on the data. DXL, DYL, and PS supervised and revised the manuscript. All authors contributed to the article and approved the submitted version.

## Funding

This research was supported by grants from the Key Projects of Medical Science and Technology in Henan Province, China No. 201602258, No. 212102310132.

## Conflict of Interest

The authors declare that the research was conducted in the absence of any commercial or financial relationships that could be construed as a potential conflict of interest.

## Publisher’s Note

All claims expressed in this article are solely those of the authors and do not necessarily represent those of their affiliated organizations, or those of the publisher, the editors and the reviewers. Any product that may be evaluated in this article, or claim that may be made by its manufacturer, is not guaranteed or endorsed by the publisher.

## Supplementary Material

Supplementary Figure 1 Scatter plot showing the count of T-CTCs **(A)**, H-CTCs **(B)**, E-CTCs **(C)**, M-CTCs **(D)** per 5 ml in PoV and PV samples. Dashed line indicates linear fit.

Supplementary Figure 2 PV CTCs counts in unresectable PDAC patients. Unresectable patients have significantly higher H-CTC count than resectable patients **(A)**. Kaplan-Meier analysis shown that there was no significant difference on overall survival time in patients with increased or non-increased T-CTCs **(B)**, E-CTCs **(C)**, H-CTCs **(D)**, M-CTCs **(E)**.

## Abbreviations

AJCC, American Joint Committee on Cancer; AUC, Area under the curve; CA 19-9, Cancer antigen 19-9; CK8/18/19, cytokeratin8/18/19; CTC, Circulating tumor cell; DAPI, 4′,6-diamidino-2-phenylindole; E-CTC, Epithelial CTC; EDTA, Ethylenediaminetetraacetic acid; EMT, Epithelial-mesenchymal transition; EpCAM, Epithelial cell adhesion molecule; H-CTC, Hybrid CTC; HR, hazard ratio; IQR, Interquartile Range; ISET, Isolation by Size of Epithelial Tumor Cells, M-CTC, Mesenchymal CTC; MFS, metastasis free survival; OS, Overall survival; PDAC, Pancreatic ductal adenocarcinoma; PoV, Portal venous; PV, peripheral PV; RFS, Recurrence free survival; ROC, Receiver operating characteristic; SD, Standard deviation; T-CTC,Total CTC.

## References

[1] Houg DS Bijlsma MF. The Hepatic Pre-Metastatic Niche in Pancreatic Ductal Adenocarcinoma. Mol Cancer (2018) 17(1):95. doi: 10.1186/s12943-018-0842-9 29903049 PMC6003100

[2] Rahib L Smith BD Aizenberg R Rosenzweig AB Fleshman JM Matrisian LM. Projecting Cancer Incidence and Deaths to 2030: The Unexpected Burden of Thyroid. Liver Pancreas Cancers United States (2014) 74(11):2913–21. doi: 10.1158/0008-5472.CAN-14-0155%J Cancer Research 24840647

[3] Groot VP Gemenetzis G Blair AB Rivero-Soto RJ Yu J Javed AA. Defining and Predicting Early Recurrence in 957 Patients With Resected Pancreatic Ductal Adenocarcinoma. Ann Surg (2019) 269(6):1154–62. doi: 10.1097/sla.0000000000002734 PMC6191366 31082915

[4] Tao L Su L Yuan C Ma Z Zhang L Bo S. Postoperative Metastasis Prediction Based on Portal Vein Circulating Tumor Cells Detected by Flow Cyt Ometry in Periampullary or Pancreatic Cancer. Cancer Manag Res (2019) 11:7405–25. doi: 10.2147/CMAR.S210332 PMC6689556 31496801

[5] Qi LN Xiang BD Wu FX Ye JZ Zhong JH Wang YY. Circulating Tumor Cells Undergoing EMT Provide a Metric for Diagnosis and Prognosis of Patients With Hepatocellular Carcinoma. Cancer Res (2018) 78(16):4731–44. doi: 10.1158/0008-5472.Can-17-2459 29915159

[6] Zhou B Xu JW Cheng YG Gao JY Hu SY Wang L. Early Detection of Pancreatic Cancer: Where Are We Now and Where are We Going? Int J Cancer (2017) 141(2):231–41. doi: 10.1002/ijc.30670 28240774

[7] Buscail E Chiche L Laurent C Vendrely V Denost Q Denis J. Tumor-Proximal Liquid Biopsy to Improve Diagnostic and Prognostic Performances of Circulating Tumor Cells. Mol Oncol (2019) 13(9):1811–26. doi: 10.1002/1878-0261.12534 PMC6717761 31216108

[8] Guo W Sun YF Shen MN Ma XL Wu J Zhang CY. Circulating Tumor Cells With Stem-Like Phenotypes for Diagnosis, Prognosis, and Therapeutic Response Evaluation in Hepatocellular Carcinoma. Clin Cancer Res (2018) 24(9):2203–13. doi: 10.1158/1078-0432.CCR-17-1753 29374055

[9] Liu X Li C Li J Yu T Zhou G Cheng J. Detection of CTCs in Portal Vein was Associated With Intrahepatic Metastases and Prognosis in Patients With Advanced Pancreatic Cancer. J Cancer (2018) 9(11):2038–45. doi: 10.7150/jca.23989 PMC5995938 29896289

[10] Tien YW Kuo HC Ho BI Chang MC Chang YT Cheng MF. A High Circulating Tumor Cell Count in Portal Vein Predicts Liver Metastasis From Periampullary or Pancreatic Cancer: A High Portal Venous CTC Count Predicts Liver Metastases (2016) 95(16)e3407. doi: 10.1097/md.0000000000003407 PMC4845834 27100430

[11] Reddy RM Murlidhar V Zhao L Grabauskiene S Zhang Z Ramnath N. Pulmonary Venous Blood Sampling Significantly Increases the Yield of Circulating Tumor Cells in Early-Stage Lung Cancer. J Thorac Cardiovasc Surg (2016) 151(3):852–8. doi: 10.1016/j.jtcvs.2015.09.126 26614417

[12] Nieto MA Huang RY Jackson RA Thiery JP. EMT: 2016. Cell (2016) 166(1)21–45. doi: 10.1016/j.cell.2016.06.028 27368099

[13] Pastushenko I Brisebarre A Sifrim A Fioramonti M Revenco T Boumahdi S. Identification of the Tumour Transition States Occurring During EMT. Nature (2018) 556(7702):463–8. doi: 10.1038/s41586-018-0040-3 29670281

[14] Pastushenko I Blanpain C. EMT Transition States During Tumor Progression and Metastasis. Trends Cell Biol (2019) 29(3):212–26. doi: 10.1016/j.tcb.2018.12.001 30594349

[15] Chen J Cao S Situ B Zhong J Hu Y Li S. Metabolic Reprogramming-Based Characterization of Circulating Tumor Cells in Prostate Cancer. J Exp Clin Cancer Res (2018) 37(1):127. doi: 10.1186/s13046-018-0789-0 29954422 PMC6025832

[16] Genna A Vanwynsberghe AM Villard AV Pottier C Ancel J Polette M. EMT-Associated Heterogeneity in Circulating Tumor Cells: Sticky Friends on the Road to Metastasis. Cancers (Basel) 12(6). doi: 10.3390/cancers12061632 PMC7352430 32575608

[17] Liu YK Hu BS Li ZL He X Li Y Lu LG. An Improved Strategy to Detect the Epithelial-Mesenchymal Transition Process in Circulating Tumor Cells in Hepatocellular Carcinoma Patients. Hepatol Int (2016) 10(4):640–6. doi: 10.1007/s12072-016-9732-7 27115761

[18] Dong J Zhu D Tang X Qiu X Lu D Li B. Detection of Circulating Tumor Cell Molecular Subtype in Pulmonary Vein Predicting Prognosis of Stage I-III Non-Small Cell Lung Cancer Patients. Front Oncol (2019) 9:e1139. doi: 10.3389/fonc.2019.01139 PMC6830362 31737568

[19] Tempero MA Malafa MP Al-Hawary M Behrman SW Benson AB Cardin DB. Pancreatic Adenocarcinoma, Version 2.2021, NCCN Clinical Practice Guidelines in Oncology. J Natl Compr Canc Netw (2021) 19(4):439–57. doi: 10.6004/jnccn.2021.0017 33845462

[20] Comprehensive Guidelines for the Diagnosis and Treatment of Pancreatic Cancer (2018 Version). Zhonghua Wai Ke Za Zhi (2018) 56(7):481–94. doi: 10.3760/cma.j.issn.0529-5815.2018.07.001 30032527

[21] Latil M Nassar D Beck B Boumahdi S Wang L Brisebarre A. Cell-Type-Specific Chromatin States Differentially Prime Squamous Cell Carcinoma Tumor-Initiating Cells for Epithelial to Mesenchymal Transition. Cell Stem Cell (2017) 20(2):191–204.e5. doi: 10.1016/j.stem.2016.10.018 27889319 PMC5939571

[22] Hong B Zu Y. Detecting Circulating Tumor Cells: Current Challenges and New Trends. Theranostics (2013) 3(6):377–94. 10.7150/thno.5195 PMC3677409 23781285

[23] Lamouille S Xu J Derynck R. Molecular Mechanisms of Epithelial-Mesenchymal Transition. Nat Rev Mol Cell Biol (2014) 15(3):178–96. 10.1038/nrm3758 PMC4240281 24556840

[24] Pastushenko I Brisebarre A Sifrim A Fioramonti M Revenco T Boumahdi S. Identification of the Tumour Transition States Occurring During EMT. Nature (2018) 556(7702):463–8. doi: 10.1038/s41586-018-0040-3 29670281

[25] Nieto MA Huang RY Jackson RA Thiery JP. EMT: 2016. Cell (2016) 166(1):21–45. doi: 10.1016/j.cell.2016.06.028 27368099

[26] Sundling KE Lowe AC. Circulating Tumor Cells: Overview and Opportunities in Cytology. Adv Anat Pathol (2019) 26(1):56–63. doi: 10.1097/pap.0000000000000217 30325755

[27] Sun Y Wu G Cheng KS Chen A Neoh KH Chen S. CTC Phenotyping for a Preoperative Assessment of Tumor Metastasis and Overall Survival of Pancreatic Ductal Adenocarcinoma Patients. EBioMedicine (2019) 46:133–49. doi: 10.1016/j.ebiom.2019.07.044 PMC6712350 31375425

[28] Szczerba BM Castro-Giner F Vetter M Krol I Gkountela S Landin J. Neutrophils Escort Circulating Tumour Cells to Enable Cell Cycle Progression. Nature (2019) 566(7745):553–7. doi: 10.1038/s41586-019-0915-y 30728496

[29] Mathias TJ Chang KT Martin SS Vitolo MI. Gauging the Impact of Cancer Treatment Modalities on Circulating Tumor Cells (CTCs). Cancers (Basel) (2020) 12(3). 10.3390/cancers12030743 PMC7140032 32245166

[30] Yang YJ Kong YY Li GX Wang Y Ye DW Dai B. Phenotypes of Circulating Tumour Cells Predict Time to Castration Resistance in Metastatic Castration-Sensitive Prostate Cancer. BJU Int (2019) 124(2):258–67. doi: 10.1111/bju.14642 30536520

[31] Dong X Ma Y Zhao X Tian X Sun Y Yang Y. Spatial Heterogeneity in Epithelial to Mesenchymal Transition Properties of Circulating Tumor Cells Associated With Distant Recurrence in Pancreatic Cancer Patients. 2020 (2020) 8(11):676. doi: 10.21037/atm-20-782 PMC7327339 32617296

[32] Wei C Yang C Wang S Shi D Zhang C Lin X. Crosstalk Between Cancer Cells and Tumor Associated Macrophages Is Required for Mesenchymal Circulating Tumor Cell-Mediated Colorectal Cancer Metastasis. Mol Cancer (2019) 18(1):64. doi: 10.1186/s12943-019-0976-4 30927925 PMC6441214

[33] Li S Chen Q Li H Wu Y Feng J Yan Y. Mesenchymal Circulating Tumor Cells (CTCs) and OCT4 mRNA Expression in CTCs for Prognosis Prediction in Patients With Non-Small-Cell Lung Cancer. Clin Transl Oncol (2017) 19(9):1147–53. doi: 10.1007/s12094-017-1652-z 28374320

[34] Kim M-Y Oskarsson T Acharyya S Nguyen DX Zhang XHF Norton L. Tumor Self-Seeding by Circulating Cancer Cells. Cell (2009) 139(7):1315–26. doi: 10.1016/j.cell.2009.11.025 PMC2810531 20064377

[35] White MG Lee A Vicente D Hall C Kim MP Katz MHG. Measurement of Portal Vein Blood Circulating Tumor Cells is Safe and May Correlate With Outcomes in Resected Pancreatic Ductal Adenocarcinoma. Ann Surg Oncol (2021) 28(8):4615–22. doi: 10.1245/s10434-020-09518-y PMC8607961 33415562

[36] Denve E Riethdorf S Ramos J Nocca D Coffy A Daurs JP. Alix-Panabires: Capture of Viable Circulating Tumor Cells in the Liver of Colorectal Cancer Patients. Clin Chem (2013) 59(9):1384–92. doi: 10.1373/clinchem.2013.202846 23695297

[37] Tien YW Kuo HC Ho BI Chang MC Chang YT Cheng MF. A High Circulating Tumor Cell Count in Portal Vein Predicts Liver Metastasis From Periampullary or Pancreatic Cancer: A High Portal Venous CTC Count Predicts Liver Metastases. Med (Baltimore) (2016) 95(16):e3407. doi: 10.1097/md.0000000000003407 PMC4845834 27100430

[38] Catenacci DV Chapman CG Xu P Koons A Konda VJ Siddiqui UD. Acquisition of Portal Venous Circulating Tumor Cells From Patients With Pancreaticobiliary Cancers by Endoscopic Ultrasound. Gastroenterology (2015) 149(7):1794–803.e4. doi: 10.1053/j.gastro.2015.08.050 26341722 PMC4985007

[39] Chapman CG Ayoub F Swei E Llano EM Li B Siddiqui UD. Endoscopic Ultrasound Acquired Portal Venous Circulating Tumor Cells Predict Progression Free Survival and Overall Survival in Patients With Pancreaticobiliary Cancers. Pancreatology (2020) 20(8):1747–54. doi: 10.1016/j.pan.2020.10.039 33082106

[40] Chapman CG Waxman I. EUS-Guided Portal Venous Sampling of Circulating Tumor Cells. Curr Gastroenterol Rep (2019) 21(12):68. doi: 10.1007/s11894-019-0733-2 31813055

[41] Tan K Leong SM Kee Z Caramat PV Teo J Blanco MVM. Longitudinal Monitoring Reveals Dynamic Changes in Circulating Tumor Cells (CTCs) and CTC-Associated miRNAs in Response to Chemotherapy in Metastatic Colorectal Cancer Patients. Cancer Lett (2018) 423:1–8. doi: 10.1016/j.canlet.2018.02.039 29518480

[42] Zhang Z Xiao Y Zhao J Chen M Xu Y Zhong W. Relationship Between Circulating Tumour Cell Count and Prognosis Following Chemotherapy in Patients With Advanced Non-Small-Cell Lung Cancer. Respirology (2016) 21(3):519–25. doi: 10.1111/resp.12696 26661896

[43] Xu Y Qin T Li J Wang X Gao C Xu C. Detection of Circulating Tumor Cells Using Negative Enrichment Immunofluorescence and an *In Situ* Hybridization System in Pancreatic Cancer. Int J Mol Sci (2017) 18(4). doi: 10.3390/ijms18040622 PMC5412265 28333072

[44] Bidard FC Huguet F Louvet C Mineur L Bouché O Chibaudel B. Circulating Tumor Cells in Locally Advanced Pancreatic Adenocarcinoma: The Ancillary CirCe 07 Study to the LAP 07 Trial. Ann Oncol (2013) 24(8):2057–61. doi: 10.1093/annonc/mdt176 23676420

[45] Chong MH Zhao Y Wang J Zha XM Liu XA Ling LJ. The Dynamic Change of Circulating Tumour Cells in Patients With Operable Breast Cancer Before and After Chemotherapy Based on a Multimarker QPCR Platform. Br J Cancer (2012) 106(10):1605–10. doi: 10.1038/bjc.2012.157 PMC3349184 22516945

