# Supplementary figures and images for: Portal Venous Circulating Tumor Cells Undergoing Epithelial-Mesenchymal Transition Exhibit Distinct Clinical Significance in Pancreatic Ductal Adenocarcinoma
Source: Front Oncol. 2021 Oct 28;11:757307. doi: 10.3389/fonc.2021.757307 (PMC8582019; doi:10.3389/fonc.2021.757307)

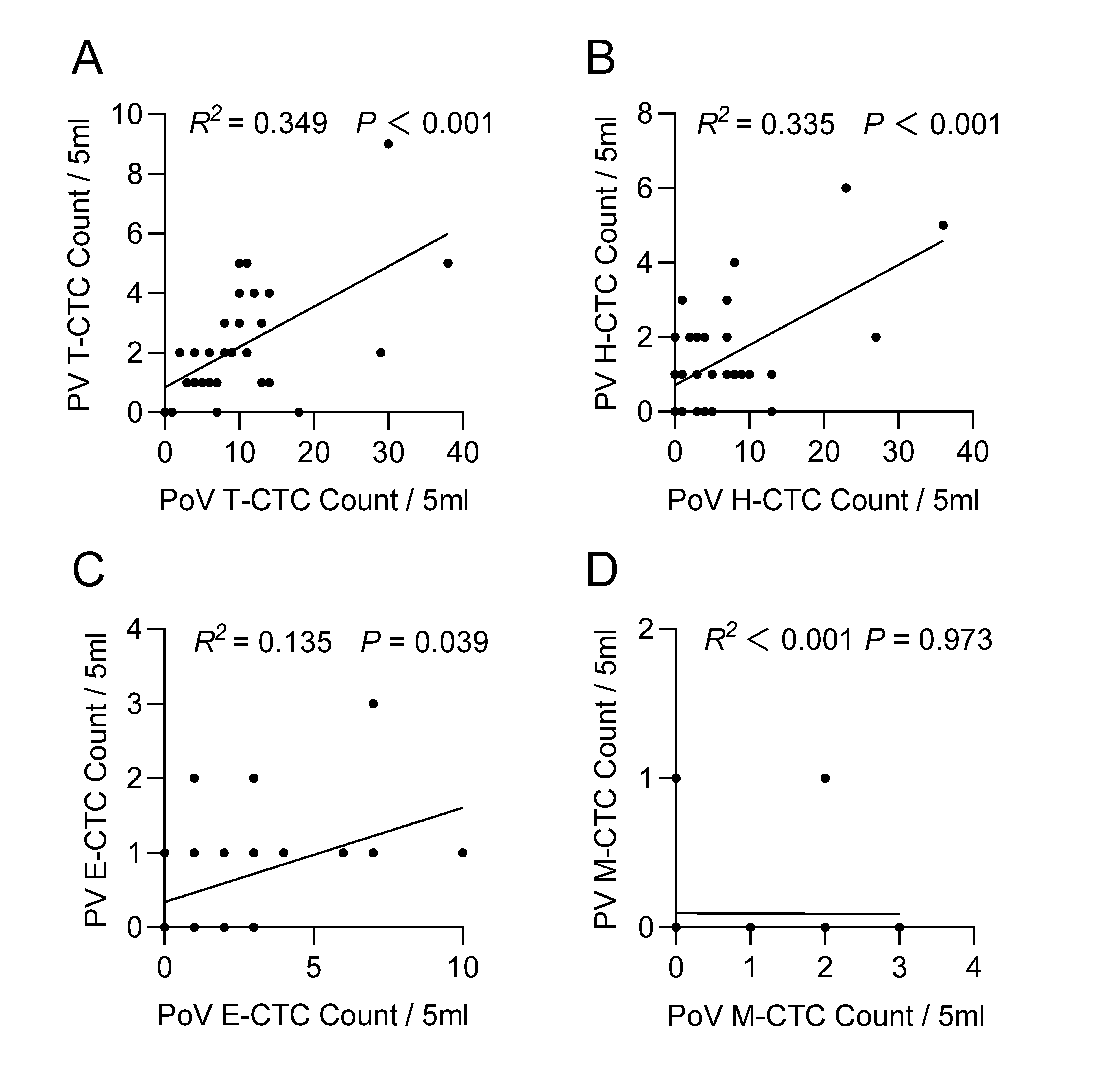

Supplement: Supplementary Figure 1 — Scatter plot showing the count of T-CTCs (A), H-CTCs (B), E-CTCs (C), M-CTCs (D) per 5 ml in PoV and PV samples. Dashed line indicates linear fit. [file Image_1.tif]
